# EEG topographies provide subject-specific correlates of motor control

**DOI:** 10.1038/s41598-017-13482-1

**Published:** 2017-10-16

**Authors:** Elvira Pirondini, Martina Coscia, Jesus Minguillon, José del R. Millán, Dimitri Van De Ville, Silvestro Micera

**Affiliations:** 1 Bertarelli Foundation Chair in Translational Neuroengineering, Center for Neuroprosthetics and Institute of Bioengineering, School of Engineering, École Polytechnique Fédérale de Lausanne (EPFL), Lausanne, 1015 Switzerland; 2 Wyss Center for Bio- and Neuro- Engineering, Geneva, 1202 Switzerland; 3 Department of Computer Architecture and Technology, University of Granada, Granada, 18071 Spain; 4 Chair in Brain-Machine Interface, Center for Neuroprosthetics, School of Engineering, École Polytechnique Fédérale de Lausanne (EPFL), Lausanne, 1015 Switzerland; 5 Medical Image Processing Lab, Institute of Bioengineering, Center for Neuroprosthetics, School of Engineering, École Polytechnique Fédérale de Lausanne (EPFL), Lausanne, 1015 Switzerland; 6 Department of Radiology and Medical Informatics, University of Geneva, Geneva, 1211 Switzerland; 7 Translational Neural Engineering Area, The Biorobotics Institute, Scuola Superiore Sant’Anna, Pisa, 56025 Italy

## Abstract

Electroencephalography (EEG) of brain activity can be represented in terms of dynamically changing topographies (microstates). Notably, spontaneous brain activity recorded at rest can be characterized by four distinctive topographies. Despite their well-established role during resting state, their implication in the generation of motor behavior is debated. Evidence of such a functional role of spontaneous brain activity would provide support for the design of novel and sensitive biomarkers in neurological disorders. Here we examined whether and to what extent intrinsic brain activity contributes and plays a functional role during natural motor behaviors. For this we first extracted subject-specific EEG microstates and muscle synergies during reaching-and-grasping movements in healthy volunteers. We show that, in every subject, well-known resting-state microstates persist during movement execution with similar topographies and temporal characteristics, but are supplemented by novel task-related microstates. We then show that the subject-specific microstates’ dynamical organization correlates with the activation of muscle synergies and can be used to decode individual grasping movements with high accuracy. These findings provide first evidence that spontaneous brain activity encodes detailed information about motor control, offering as such the prospect of a novel tool for the definition of subject-specific biomarkers of brain plasticity and recovery in neuro-motor disorders.

## Introduction

A large body of neuroimaging^1–3^ and computational^4,5^ research has revealed the complexity and richness of spontaneous brain activity measured at rest. It is now widely acknowledged that spontaneous brain activity is not just “noise”, but exhibits distinct spatiotemporal organization at the level of large-scale distributed neuroanatomical systems^6^. However, whether and to what extent spontaneous brain activity is modified to support behaviors such as sensory-motor tasks is still unclear^6,7^. Highlighting a functional role of the resting-state activity would be of great clinical value, potentially providing novel, rich, and sensitive biomarkers for neurological disorders, which impair patients’ ability to perform sensory-motor tasks^8^. These biomarkers could maximize therapeutic effects by informing personalization of therapy selection, timing, and duration^9^. However, mechanisms of neuronal reorganization and plasticity are inconsistent and variable at the individual level^8^ and, thus, their investigation entails the definition of subject-specific biomarkers. Therefore, the use of spontaneous brain activity as a biomarker of recovery mechanisms and neural deficits in neuro-motor disorders is contingent on the demonstration that: 1) this intrinsic activity plays a functional role in motor behaviors, and 2) that this relation with sensory-motor tasks can be extracted in each subject independently.

When recorded with electroencephalography (EEG), spontaneous brain activity can be represented in terms of microstates, which are brain states characterized by periods of coherent and synchronized neural activation and marked by distinctive topographies of the scalp electrical potential^10–12^. Prototypical topographies with typical durations of 50–150ms have been associated with four states that are persistently observed across the entire human life span^10^. They have been attributed functional relevance as well as been related to some functional magnetic resonance imaging (fMRI) resting-state networks (RSNs)^11,13–15^.

Here we tested whether subject-specific resting-state activity persists during volitional movements, how it is modified and supplemented, and whether it is predictive of motor behavior.

For each of the enrolled healthy volunteers, we extracted EEG microstates independently during upper limb movement planning and execution. Simultaneously, we recorded high fidelity (3 kHz) wireless electromyographic (EMG) activity of 15 arm and hand muscles to extract muscle synergies^16–18^ and investigate the correlation between these signals and the brain activity in each individual. Muscle synergies obtained from the decomposition of the EMG signals have been extensively proposed to study muscle coordination and motor control strategies^19–25^. Moreover, cortical motor neuron activity seems to encode the recruitment of motor primitives in the form of spatiotemporal muscular and kinematic synergies^26–28^.

We first compared individual microstates that emerged at rest against those extracted during motor tasks^29^. We found that some of the resting-state microstates persisted during motor behavior, but the set of brain states was supplemented with task-specific microstates as well. Second, we explored the correlation between temporal dynamics of subject-specific microstates and the emergence of muscle synergies during movement using multivariate analysis. We show that muscle synergies activation was correlated with microstates’ dynamical organization in each of the enrolled subjects. Finally, we used the subject-specific microstates occurrence to decode individual grasping movements with high accuracy in all subjects, demonstrating high degree of correlation with specific motor-tasks.

The preservation and correlation of spontaneous brain activity with task-specific motor behaviors and the corroboration of this evidence in all subjects highlight its potential to study brain plasticity and recovery processes in neuro-motor disorders.

## Results

In the experiment, subjects were asked to perform pure planar reaching and reaching-and-grasping movements while high-density EEG and EMG activity was acquired. Subject-specific EEG microstates were extracted for each individual for each motor task and for resting state separately. They were identified as the best solution of the cluster analysis using cross-validation without any a priori assumptions on the number of clusters or on the minimum explained variance. Subject-specific microstates were then compared across conditions and their dynamics was correlated with muscle synergies.

### Resting-state microstates are present during motor tasks

**Figure 1:**
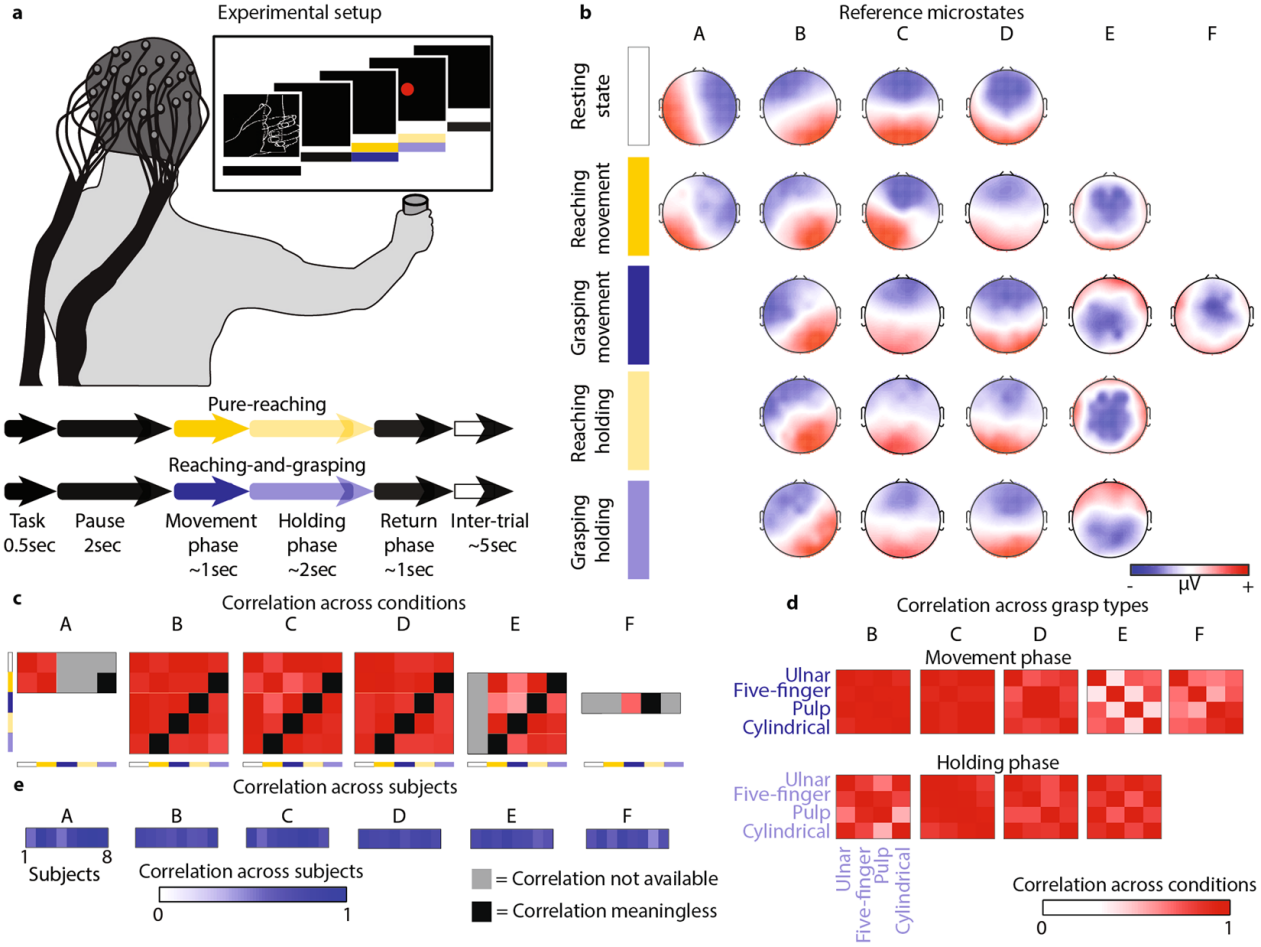
EEG microstates. Subjects were asked to execute pure planar reaching movements or to reach, grasp, and hold 16 different objects with four different grasp types. A screen was used to indicate the experimental timeline. (**a**) Subject-specific EEG microstates were extracted for each subject and dataset using CARTOOL^77^. The plot reports typical subject-specific microstates for resting state (white bar – first row), pure-reaching during movement phase (yellow bar – second row), reaching-and-grasping during movement phase (blue bar – third row), pure-reaching during holding phase (light yellow bar – fourth row), and reaching-and-grasping during holding phase (light blue bar – fifth row). Red and blue colors correspond to positive and negative voltages, respectively. (**b**) To identify the presence of spontaneous brain activity during motor behavior, subject-specific microstates found during motor task conditions were matched to the resting-state microstates. The non-matching microstates were then compared across motor task conditions. Correlation across conditions (**c**) and across grasp types (**d**) are reported coded in red for each microstate. Correlations are reported in absolute values. Grey squares code “correlation not available” (e.g., for microstate E the comparisons with the resting-state condition are not possible because microstate E is not present during resting state). Black squares code “correlation meaningless”. Indeed, we considered meaningless to compare the holding phase of a specific motor task with the movement phase of another motor task (e.g., the holding phase of pure-reaching with the movement phase of a grasp type). For the purpose of summarizing the results across all subjects, subject-specific microstates were matched across individual using a second k-means cluster analysis. For each subject and microstate, averaged correlation values across conditions are reported coded in blue. (**e**) Correlation values were calculated between the maps presenting highest similarity within a cluster and the subject-specific microstates within the same cluster.

During resting state, we found a set of four microstates (A, B, C, and D; mean of optimal subject’s clusters: 4; range [2 5]) consistent across subjects (R = 0.79, Fig. 1e) and in agreement with extensive literature^10,11,30^ (Fig. 1b). During all the volitional motor tasks, instead, we identified up to five EEG microstates (Fig. 1b, mean across subjects: 5; range [3 7]; consistency across subjects: R = 0.68 and R = 0.76 for pure-reaching and reaching-and-grasping, respectively, Fig. 1e).

Interestingly, four of the EEG microstates related to pure-reaching movements were highly correlated to those of the resting state, supporting the hypothesis that states of ongoing spontaneous activity are also commonly active during behavioral tasks^31–33^ (R > 0.71 for microstates A, B, C, and D, Fig. 1b,c). Reaching-and-grasping movements showed similar results albeit they did not present microstate A (R > 0.66 for microstates B, C, and D and R = 0.49 for microstate A, Fig. 1b,c).

The remaining microstates (E and F, mean correlation with microstates A, B, C, and D of the resting state was 0.38 and 0.36, respectively for E and F, Fig. 1c), whose activation was maximum over the central electrodes, were either task-specific (microstate E, R = 0.41 between pure-reaching and reaching-and-grasping movements and R = 0.52 across grasp types, Fig. 1b–d) or grasping-specific (microstate F, mean R = 0.75 across grasp types and non-present during pure-reaching movements) revealing a different topological organization not only across tasks but also across grasp types.

During the holding phase, the microstates repertoire was characterized by four states as in the resting-state condition (Fig. 1b, mean across subjects: 4; range [3 5]; consistency across subjects: R = 0.64 and R = 0.75 for pure-reaching and reaching-and-grasping, respectively, Fig. 1e). Similarly to movement phase, three of these microstates (B, C, and D) correlated with those of the resting state (R > 0.79 for pure-reaching and R > 0.73 for reaching-and-grasping, Fig. 1c). However, it should be noticed that in the case of pure-reaching during the holding phase the microstate D correlates more with the resting-state microstate C (0.87) than with the resting D (0.79), this could be caused by the similarity in the topography of these maps. The fourth microstate, instead, correlated with the task-specific microstate (i.e., E, R = 0.87 for pure-reaching and R = 0.64 for reaching-and-grasping, Fig. 1c,d) and was similar across tasks (mean R = 0.83 between pure-reaching and reaching-and-grasping movements and mean R = 0.78 across grasp types) emphasizing a topological organization across tasks and grasp types more similar during the holding phase than during movement execution.

### Muscle synergy analysis allow discriminating motor control strategies across motor task

Several studies have proposed that the motor commands for limb movements’ generation originate from a small set of motor primitives termed muscle synergies, which simultaneously recruit sets of muscles reducing the redundant degrees of freedom of the human body^16–18,34,35^. Muscle and kinematic synergies related to arm and hand motor tasks seem to be encoded in the cortex^26–28^. We, thus, reasoned that muscle synergies were an elegant tool to analyze differences in motor control strategies across multiple motor tasks. Muscle synergies were extracted using L2-norm non-negative matrix factorization for each subject independently^36^.

The cortical activity during the holding phase was characterized by a reduced complexity with respect to movement execution, i.e., reduced number of microstates and more similar topological organization across tasks. We thus hypothesized a similar behavior in motor control strategies. Indeed, five (5.26 ± 0.35 across subjects and motor tasks) and four (4.3 ± 0.43) temporal activation profiles coalescing weighted combinations of the recorded muscle activity were sufficient to reconstruct more than 98% of the variance in the original signals respectively for movement execution and holding phase, thus, confirming our hypothesis (Supplementary Figure S2a).

**Figure 2:**
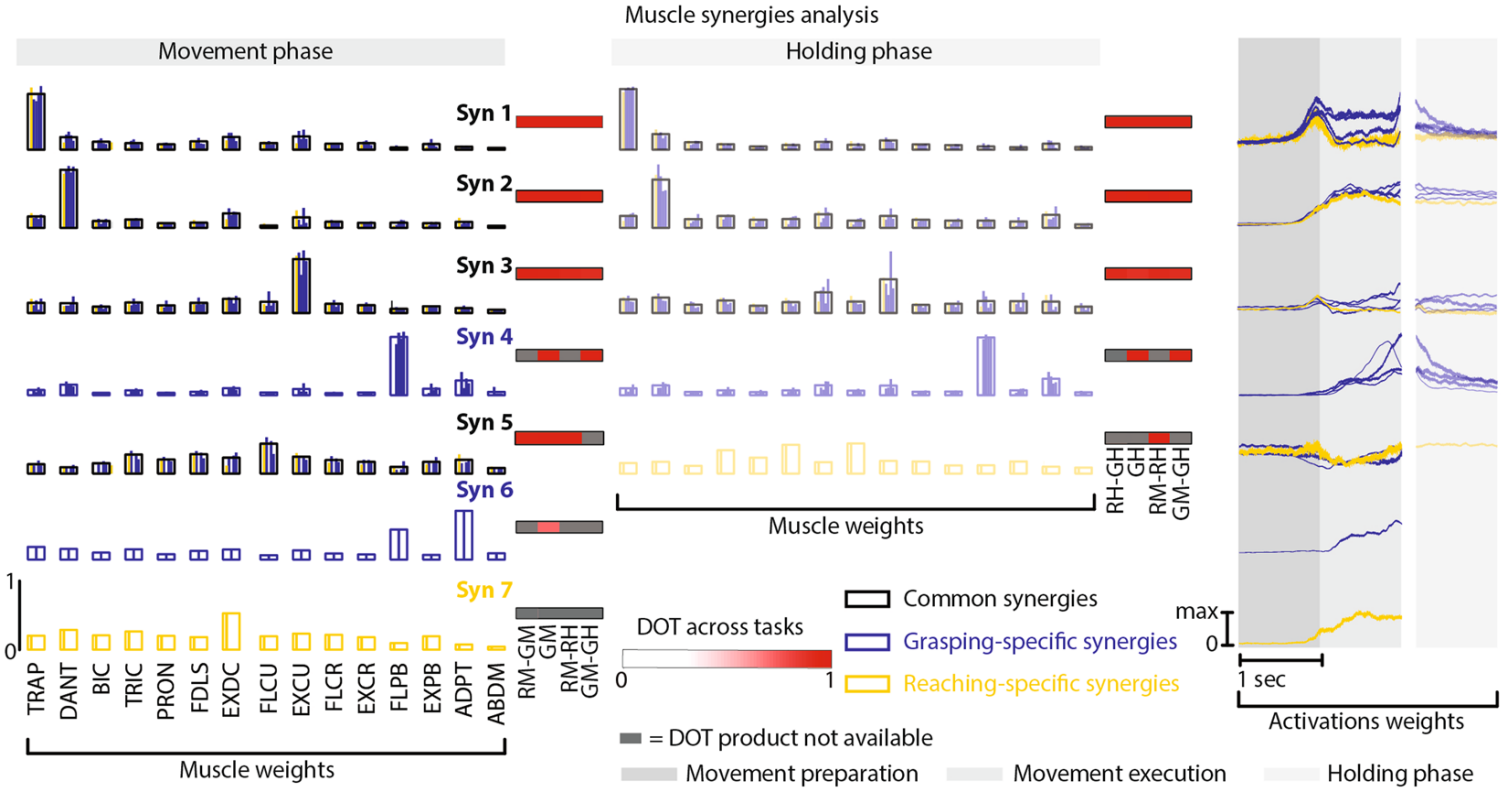
Muscle synergies. Subject-specific muscle synergies were extracted using the L2-norm non-negative matrix factorization algorithm^36^ for each subject and motor task independently. Muscle synergies were matched among subjects and conditions according to their similarity with a set of reference synergies. Left panels: muscle weights vectors for each reference synergy during movement phase. Central panels: muscle weights vectors for each reference synergy during holding phase. For synergies common across motor tasks (i.e., Syn 1, 2, 3, and 5), blue and yellow bars show the weight coefficients for each grasp type (blue bars) and for pure-reaching (yellow bar). Black bar profiles indicate means across motor tasks. For grasping-specific synergies (i.e., Syn 4 and 6), blue bars show the weight coefficients for each grasp type and blue bar profiles indicate means across grasp types. DOT values across motor tasks are reported for each synergy using red levels: RM-GM and RH-GH are the DOT products between pure-reaching and reaching-and-grasping during movement and holding phase, respectively. GM and GH are the average DOT products across grasp types during movement and holding phase, respectively; RM-RH and GM-GH are the DOT products between movement and holding phase for pure-reaching and reaching-and-grasping, respectively. RM indicates pure-reaching during movement phase; GM indicates reaching-and-grasping during movement phase; RH indicates pure-reaching during holding phase; GH indicates reaching-and-grasping during holding phase. Grey squares code “DOT product not available” (e.g., for Syn 4 comparisons between reaching-and-grasping and pure-reaching is not possible because Syn 4 was not present during pure-reaching). Right panels: muscle activation coefficients vectors for each muscle synergy during movement phase (left panels) and holding phase (right panels). Muscle activation coefficients vectors were normalized by their maximum for each muscle synergy and motor task separately.

The first (Syn 1) and the second (Syn 2) synergies enabled resistance to gravity and produced upper-limb extension (Fig. 2). These synergies together with the third synergy (Syn 3), which promoted finger extension, were common across motor tasks (mean DOT = 0.91). The fourth synergy (Syn 4), instead, was grasping-specific and dedicated to the control of the thumb (mean DOT = 0.97 across grasp types and mean DOT = 0.41 between pure-reaching and reaching-and-grasping). Indeed, pure-reaching movements did not require a fine control of the fingers, and thus Syn 4 was substituted by a synergy (Syn 7) that contributed to the extension of upper and forearm. Surprisingly the fifth synergy (Syn 5), which represented the contribution of the finger flexors and was not present in the holding phase except in pure-reaching, was substituted in five-finger pinch by an additional synergy (Syn 6) for the control of the thumb (mean DOT = 0.62).

Whereas synergies 3 and 5 had a tonic activation during movement preparation and execution and can thus be considered postural synergies; synergies 1, 2, 4, 6, and 7 emerged during the movement phase. Timing activations were in general similar across motor tasks (R = 0.75 and R = 0.48 across grasp types and R = 0.68 and R = 0.34 between pure-reaching and reaching-and-grasping for movement and holding phase, respectively, Supplementary Figure S2b).

These analyses uncovered the function and temporal structure underlying the successive activation of the motor primitives during upper-limb movements highlighting differences across motor tasks and allowing the identification of a possible link with the temporal organization of the EEG microstates.

### Resting-state microstates correlate with beta modulation

We verified that the signals used to extract the EEG microstates during volitional movements contained features commonly identified as characteristic of motor-related brain activity (i.e., beta modulation before movement initiation and after object grasp^37,38^), and we tested whether these features correlated with microstates.

**Figure 3:**
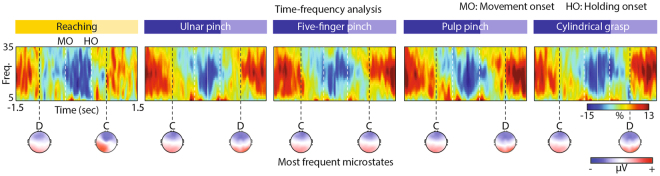
Time-frequency analysis. Time frequency spectra were calculated for each subject, epoch, and motor task from 1.5 second before movement onset (MO) to 1.5 second after object grasp (i.e., holding onset, HO). The plots display power spectra averaged across epochs and subjects and across 7 electrodes of interest (Cz, CPz, Pz, C1, C3, C2, C4) for each motor task separately. The power spectrum is expressed in percentage compared to the average spectrum (0%) as a function of time. White dashed lines code movement (MO) and holding onset (HO). Black dashed lines code specific time point of beta-desynchronization and beta-rebound onset. Most frequent microstates across epochs and subjects occurring at the time point of beta-desynchronization/rebound onset (identified from the averaged time frequency spectra over the 7 electrodes of interest) are reported in correspondence of the black dashed lines. The most frequent microstates were estimated counting the number of times (i.e., across epochs and subjects) each microstate was found at the specific time points of beta-desynchronization and beta-rebound. Red and blue colors correspond to positive and negative voltages, respectively.

The EEG data during movements showed characteristic beta oscillation modulations in agreement with previous results (Fig. 3)^37,38^. We observed an increase in the beta band as early as 1.5 seconds before movement initiation with a centro-parietal distribution (see Supplementary Figure S3). From 0.70 ± 0.10 seconds before movement onset, a decrease in the beta power gradually emerged, reaching its maximum around movement onset. Finally, a beta-rebound occurred from 0.68 ± 0.13 seconds after object grasp. No differences in the beta power modulations were identified across motor tasks in agreement with previous studies^37^. The lack of differences, already reported^37^ and in contrast with earlier studies on monkey local field potentials^39^ and on electrocorticography recordings^40^, could be attributed to the resolution of the EEG recordings.

Interestingly, the most frequent microstates at the specific time points of beta-desynchronization and beta-rebound were resting-state microstates. In particular, for ulnar pinch, pulp pinch, and cylindrical grasp, the most frequent microstates at the time points of the beta-desynchronization and beta-rebound were C and D, respectively, as opposed to pure-reaching and five-finger pinch (Fig. 3).

### Subject-specific microstates temporal dynamics contains motor information

**Figure 4:**
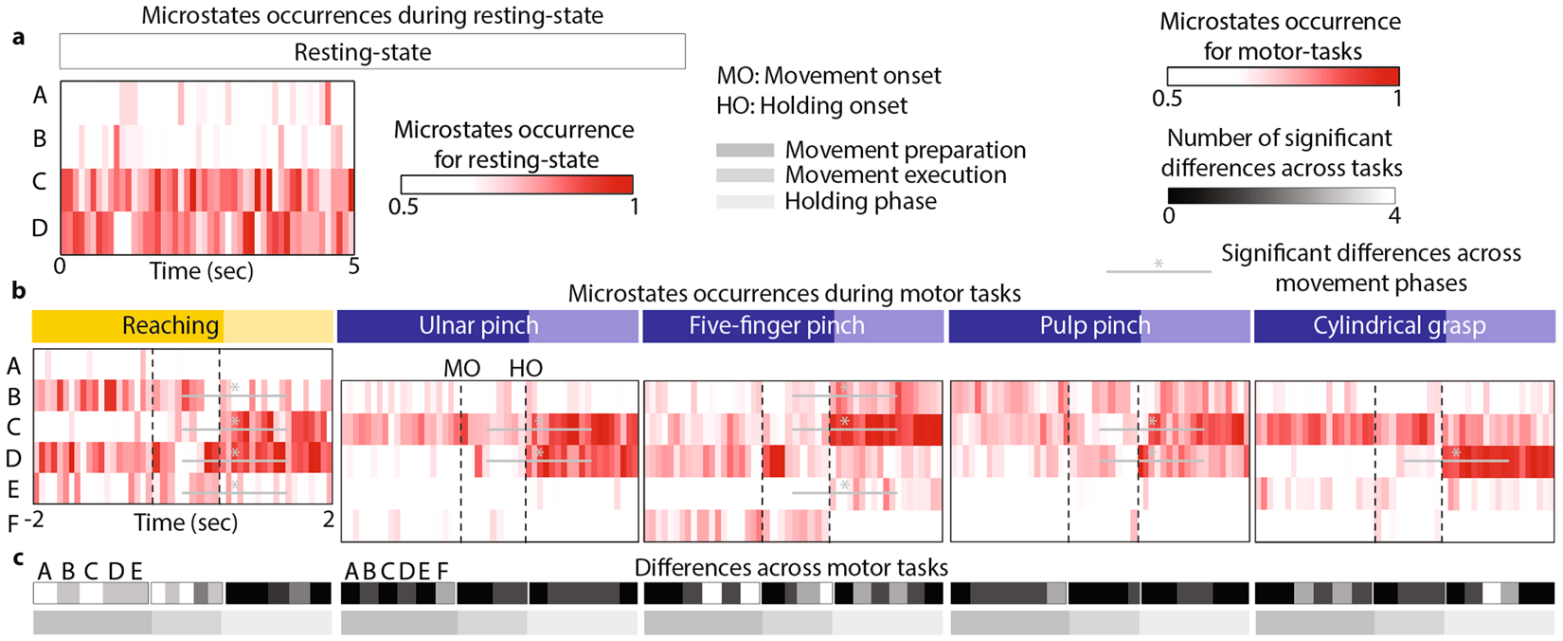
EEG microstates dynamics. We evaluated EEG microstate dynamics by computing EEG microstates occurrences for each subject independently. (**a**) EEG microstates occurrence for 5 seconds resting state averaged over subjects coded in red (range [0.5 1], see Supplementary Figure S4b for range [0 1] and S4c for statistical quantification of EEG microstates dynamics during resting state). We chose to represent 5 seconds in order to have the same temporal resolution of the motor tasks. The values of the histograms are normalized over time and microstates. (**b**) Average (across subjects) EEG microstates occurrences for pure-reaching and each grasp type separately are coded in red (range [0.5 1], see Supplementary Figure S4a for range [0 1]). The values of the histograms are normalized over time and microstates. Black dashed lines code movement (MO) and holding onset (HO). We calculated significant differences across motion phases (i.e., between movement preparation and movement execution, and between movement execution and holding phase). Significant differences (permutation test α = 0.05) are represented with grey lines and asterisks (*). We calculated also significant differences across motor tasks for movement preparation, movement execution, and holding phase separately (**c**). For each motor task and microstate, grey levels (range [0 4], where four means that that particular motor task is significantly different than all the other four motor tasks) code the number of significant differences across motor tasks.

Despite the temporal characteristics of the microstates did not differentiate between resting-state and motor tasks (Supplementary Figure S1), the microstates dynamics could differ across conditions underlying different motor processes. Indeed, microstates dynamics have shown to correlate with altered brain functions^30,41–45^. Hence, we analyzed microstates dynamics by computing the normalized histogram of occurrence of each state during resting state and during the three motion phases: preparation, execution, and holding phase (Fig. 4).

At rest, microstates occurrence did not show any apparent modulation (Fig. 4a). Microstates C and D were dominating, but their presence was not modulated over time (see Supplementary Figure S4c). Instead, during motor tasks, microstates occurrence was temporally modulated between motion phases (Fig. 4b). Microstates C and D occurrence significantly increased (permutation test, α = 0.05) during the holding phase for all motor tasks but cylindrical grasp (for microstate C) and five-finger pinch (for microstate D). In pure-reaching movements, microstates B and E were also modulated showing a significant decrease in their occurrence rate during the holding phase. Occurrence of microstates B and E, instead, significantly increased during the holding phase of five-finger pinch.

As expected from the differences in muscle synergies, we found statistically significant differences in microstates occurrences between pure-reaching and reaching-and-grasping movements, in particular during movement preparation and execution. Microstate C was significantly more present in reaching-and-grasping movements than in pure-reaching movements during movement preparation and execution (permutation test, α = 0.05, Fig. 4c). The presence of microstates B and E, instead, was significantly higher in pure-reaching movements as compared to reaching-and-grasping. Moreover, when comparing across grasp types, we found that during five-finger pinch the microstates were differently modulated than during the other grasp types. Indeed, during movement preparation and execution five-finger pinch had a significantly reduced occurrence of microstates D and F as compared to the other motor tasks.

### Resting-state microstates correlate with muscle synergies in all subjects

Microstates occurrences were different across motor tasks (Fig. 4). We here hypothesized that this difference underlies a functional role of the resting-state activity as measured by EEG microstates in motor control. We tested our hypothesis by measuring the correlation between microstate and muscle synergies occurrence.

**Figure 5:**
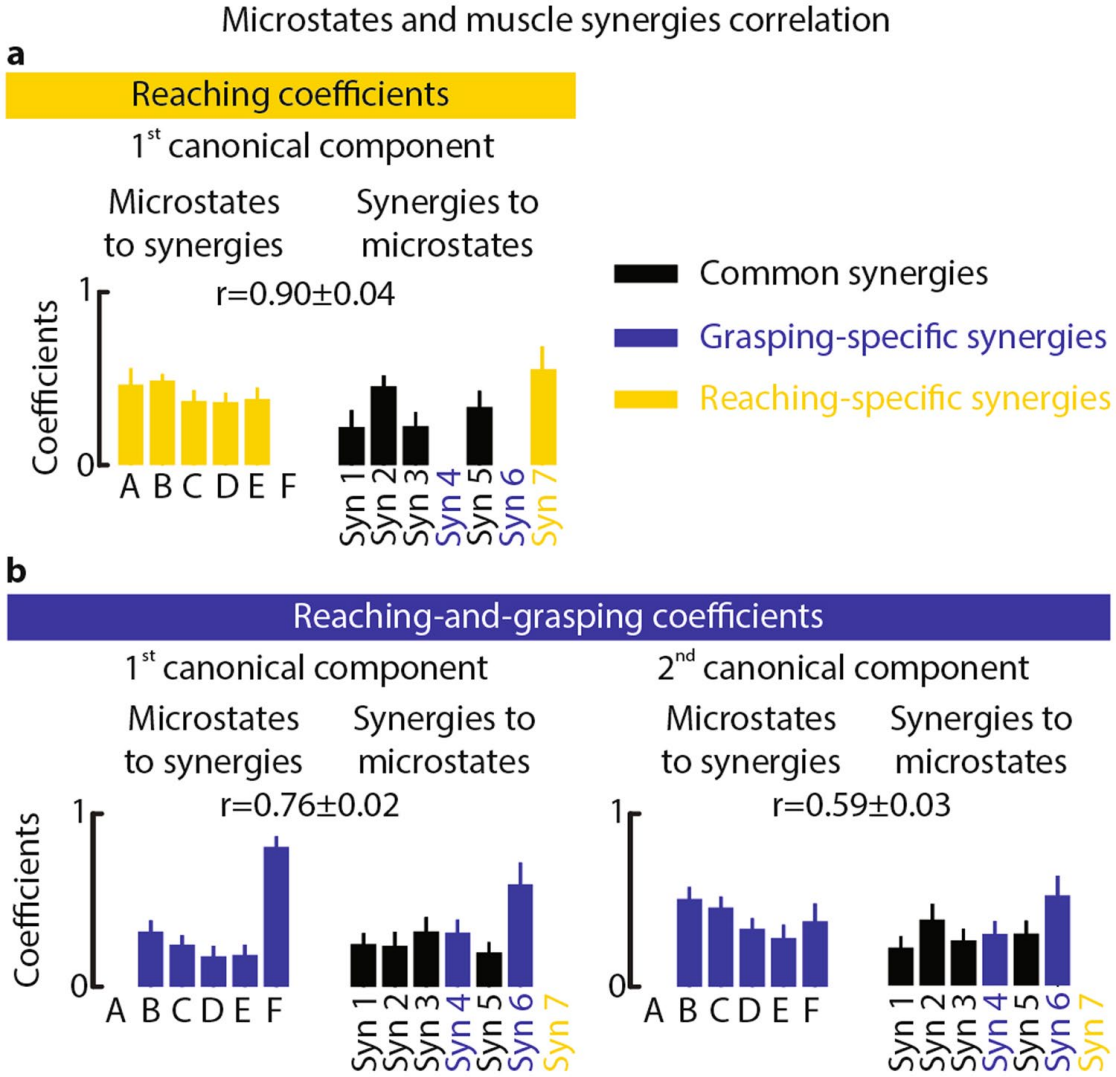
Correlation between microstates and muscle synergies. To explore the correlation between temporal dynamics of subject-specific microstates and the emergence of muscle synergies during movement, we calculated canonical correlation between all elements of the set of microstates and those of the muscle synergies set. The bar plots report average and standard error (computed over subjects) of the canonical correlation coefficients for microstates (right) and muscle synergies (left) for the first canonical component of pure-reaching (**a**). Canonical coefficients for microstates and muscle synergies were normalized by their norm before averaging them across subjects. *r* indicates average and standard error (computed over subjects) of the correlation value between microstates and muscle synergies for the statistically significant canonical component. (**b**) The bar plots report average and standard error (computed over subjects) of the canonical correlation coefficients for microstates and muscle synergies for the first canonical component (left) and the second canonical component (right) for reaching-and-grasping. *r* indicates average and standard error (computed over subjects) of the correlation value between microstates and muscle synergies for the two statistically significant canonical components. As reported in Fig. 2, synergies 1, 2, 3, and 5 are common synergies across motor tasks (represented in black). Synergies 4 and 6, instead, are grasping-specific synergies (represented in blue), and synergy 7 is reaching-specific (represented in yellow).

To compute the correlation between all elements of the set of microstates against those of the muscle synergies set, we adopted a multivariate analysis method: the canonical correlation^46^. For pure-reaching, we found one significant canonical component (p = 0.02, average correlation: r = 0.90 ± 0.04). The microstates coefficients of this component were similar for resting-state and task-related microstates, while the muscle synergies coefficients were higher for Syn 2, which produced upper-limb extension, Syn 5, which represented the contribution of the finger flexors, and Syn 7 (i.e., pure-reaching specific synergy, see Fig. 5a). For reaching-and-grasping, instead, we found two significant canonical components (p = 0.008, average correlation: r = 0.76 ± 0.02 and r = 0.59 ± 0.03, respectively, for first and second component). The first component had higher coefficients for the grasping-specific microstate (F) and for the synergies involved in the control of the fingers extension and the control of the thumb (Syn 3, Syn 4, and Syn 6, see Fig. 5b left panel). The second component, instead, had a structure similar to the component of pure-reaching. Although the coefficients for microstates D and E were lower, over all, the weights for resting-state and task-related microstates were similar. The muscle synergies coefficients, instead, were higher for Syn 2 and Syn 6, which was grasping-specific (see Fig. 5b right panel).

Despite few differences in the weight coefficients, the canonical correlation coefficients of resting-state and task-related microstates with muscle synergies activation were similar (Fig. 5). This highlights equivalent importance of both microstates sets in the generation of volitional movements.

### Subject-specific spontaneous brain activity is predictive of motor behavior

The correlation between the microstate temporal dynamics and the muscle synergies activation for each subject (Fig. 5) suggests that rich information about motor behavior is encoded in the microstates time-dependent distribution.

**Figure 6:**
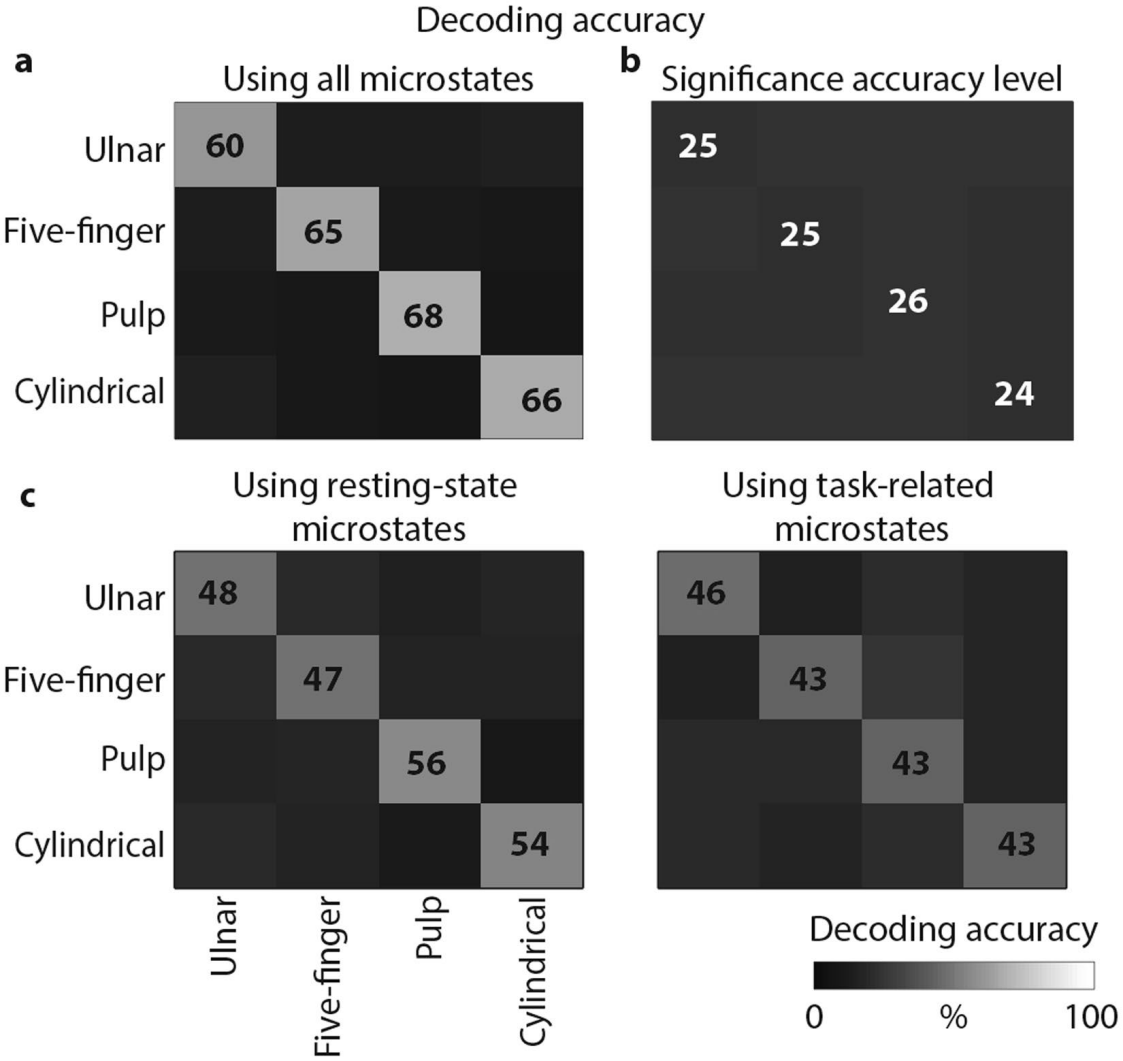
Average decoding accuracy. We employed Linear Discriminant Analysis (LDA) to further reveal the unique correspondence between microstates occurrence and motor task performed. Confusion matrices for the four grasp types were averaged over subjects and cross-validation repetitions. (**a**) Grey levels ([0 100%]) code the decoding accuracy values. (**b**) Confusion matrix for significance accuracy level. (**c**) Left: confusion matrix for the four grasp types when using only the resting-state microstates (i.e., microstates B, C, and D) occurrence over movement preparation as feature vectors. Right: confusion matrix for the four grasp types when using only the task-related microstates (i.e., microstates E and F) occurrence over movement preparation as feature vectors. In both cases, before performing LDA the influence of the occurrence of a microstates set (i.e., resting-state or task-related microstates set) was removed from that of the other set using linear regression.

**Figure 7:**
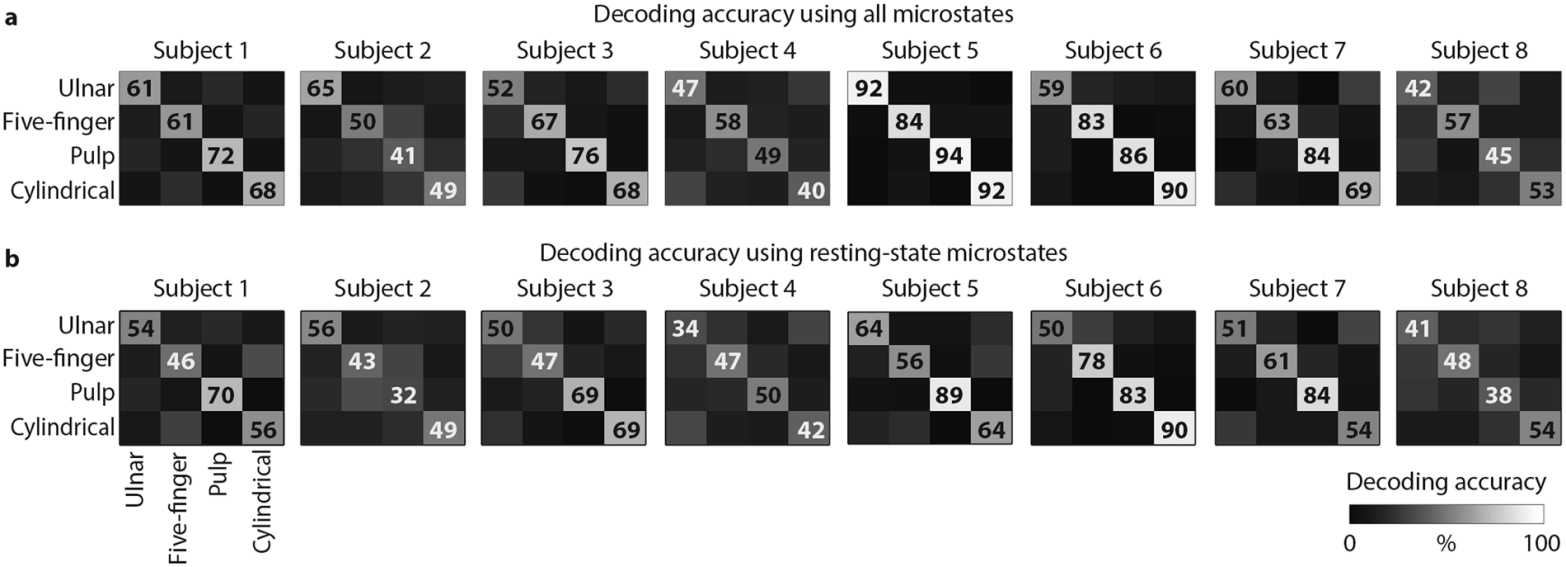
Decoding accuracy for individual subject. Confusion matrices for the four grasp types for each subject separately. For each subject, the confusion matrices were averaged over cross-validation repetitions. (**a**) The LDA was computed using both resting-state and task-related microstates. Grey levels ([0 100%]) code the decoding accuracy values. (**b**) Confusion matrix for the four grasp types obtained for each subject separately when using only the resting-state microstates (i.e., microstates B, C, and D) occurrence over movement preparation as feature vectors. Before performing LDA the influence of the occurrence of the task-related microstates set was removed from that of the resting-state microstates set using linear regression. The significant accuracy level is the same reported in Fig. 6.

To verify this hypothesis we performed a linear discriminant analysis that quantified the encoding of each grasp type in the subject-specific microstates time-dependent distribution (Fig. 6a). Using only the microstates occurrences during movement preparation as information, the LDA classifier was able to significantly distinguish between grasp types with a high decoding accuracy stable over cross-validation repetitions (65 ± 10%, with chance level of 25%, see Fig. 6b). The classifier confusion matrix was diagonal demonstrating high linear correlation between EEG microstates organization and specific motor tasks. A reduced, but still statistically significant, accuracy was achieved when using only the resting-state or the task-related microstates. However, decoding accuracy was slightly higher for resting-state microstates revealing a pivotal role of these brain states during volitional movements (average accuracy: 51% when using only resting-state microstates and 44% when using only task-related microstates, Fig. 6c). These results were reproduced in all eight subjects (Fig. 7).

These results support our hypothesis that spontaneous brain activity is present during motor-behavior and its dynamical organization encodes critical information about task execution at individual level.

## Discussion

Here, we introduced a multi-modal experimental framework employing high-density EEG and muscle synergies analysis to uncover the functional role of spontaneous brain activity during the execution of volitional arm and hand movements. We utilized subject-specific EEG topographies and muscle synergies offering as such the prospect of a novel tool to study mechanisms of neuronal reorganization and recovery processes in neuro-motor disorders.

We here discuss our results with an emphasis on the modular organization of the brain activity during resting state and volitional movements, its correlation with motor task features, and its subject-specific relation to motor control strategies. Finally, we highlight the importance of these results in the debate about the relationship between rest and task brain states, and their relevance for the definition of personalized biomarkers for clinical applications.

Based on previous findings that reported correspondence between coherent patterns of resting-state and task-related brain networks^8^, we expected that spontaneous brain activity was present during volitional movements and was predictive of motor behavior. Subject-specific microstate analysis during reaching-and-grasping movements confirmed our hypothesis. Indeed, the prototypical resting-state microstates extensively reported in literature^10,11,30^ were still present during movement execution (Fig. 1) with preserved temporal characteristics (Supplementary Figure S1). Only microstates C and D showed a reduced frequency of occurrence during the movement phase, in agreement with the work of Milz and colleagues^47^.

During movement execution, some of the resting-state microstates were substituted by two novel states (E and F). Microstate E exhibited a marked correlation with each task; whereas microstate F was specific for the execution of grasping movements, i.e., it was only present during the movement phase of reaching-and-grasping tasks (Fig. 1).

Overall, these results reveal that the brain activity during the execution of volitional movements evolves through a set of spatiotemporal states composed of resting-state patterns, with similar spatiotemporal structure, and of movement-specific patterns, which differentiated across motor tasks. Interestingly, these differences decreased during the holding phase, which required simpler motor control strategies similar for all grasps (Fig. 2) suggesting a link between motor control strategies and brain topographies organization.

In order to verify this hypothesis, we extracted muscle synergies using non-negative matrix factorization for each subject independently^36^. Indeed, muscle synergies obtained from the decomposition of the EMG signals have been extensively proposed to study motor control strategies^19–25^.

We found that reaching-and-grasping movements involve the sequential activation of extensors and flexors muscles synergies different for upper and forearm limb (Fig. 2). These results are highly consistent with previous works that reported 3–4 muscle synergies, accounting for 80–90% of the dataset variability, during multi-digit grasping movements^48,49^, which seem to be characterized by motor units synchrony in particular across extrinsic thumb and finger flexors^50^. Interestingly, the motor primitives differentiate across grasp types particularly for five-finger pinch, which required a finer control of the thumb.

A first evidence of whether spontaneous brain activity correlates to features commonly associated to motor-information content can be provided by analyzing the frequency components of the EEG signals.

By looking at the well characterized behavior of the beta-band activity during movement, we found that beta-desynchronization and rebound during movement execution and after object grasp^37,38^ (Fig. 3) were mostly associated to the presence of microstate C (beta-desynchronization) and D (beta-rebound).

This suggests the existence of a non-trivial relation between occurrences of resting-state microstates and execution of motor tasks that might be explained by beta tuning in the ventral areas of the thalamus, which are known to project to the sensorimotor cortex^51^, and that correlated with the appearance of these states^52^.

We further validated this insight by analyzing histograms representing microstate occurrences during phases of motor tasks (Fig. 4). This analysis showed strong modulation of microstate dynamics during motor phases that additionally differentiated across motor tasks in particular during movement preparation and execution.

Taken together, these results hint at the possibility that spontaneous brain activity is linked to the basic neural mechanisms underlying skilled motor control. To verify this hypothesis we analyzed the correlation between the time-dependent distribution of brain states and muscle synergies activation in all subjects independently. This analysis revealed a correlation between both resting-state and task-related microstate occurrences and task-specific muscle synergies (Fig. 5).

This correlation was not restricted to specific states, but involved the entire set of microstates suggesting that detailed motor information is encoded in the sequence of subject-specific brain states activation.

We confirmed this hypothesis by showing in all subjects that resting-state and task-related brain states allowed for successful discrimination of individual grasping movements using only the microstate occurrences during movement preparation as information^53^ (Figs 6 and 7). Moreover, we were able to accurately predict the grasp type that was executed based on the occurrences of the resting-state microstates only. Although it is not possible to determine whether the microstate dynamics encode or decode movements, this accurate discrimination implies that resting-state microstates dynamics contain information—directly or indirectly—about the motor tasks executed.

We can therefore conclude that, despite the involvement of two novel states, the resting-state topographies remained active with similar spatiotemporal characteristics during movement execution and their modulation was task-specific in each subject. These results provide significant evidence about a possible role of spontaneous brain activity into the generation and organization of the brain activity underlying the control of motor behaviors.

Two hypotheses about the relation between rest and task states have been previously proposed^6,7^. In the first hypothesis, RSNs represent a state of “idling” of the brain that dynamically reorganizes to support task performance leading to the formation of novel task networks^6,7,32,54^. In the second hypothesis, instead, the RSNs, due to their similarity in the topography^32,55,56^, are considered “priors” for task networks and are, thus, preserved during behavioral tasks.

Our extended EEG microstates repertoire, with preservation of three resting-state microstates and replacement of one microstate, together with the shared information content between resting-state and task-related microstates (Fig. 6) is consistent with both hypotheses showing that spontaneous brain activity is in part a “prior” and in part dynamically reorganized for the accomplishment of the task. However, this conclusion of simultaneous “idling-priors” states would require a stronger demonstration of the exact link between EEG microstates and large-scale brain networks. Despite previous studies conducted using magnetoencephalography (MEG)^57–59^ showed that large-scale brain networks can be detected with electrophysiological techniques such as MEG and EEG, their correspondence with EEG microstates is still unclear. Indeed, the number of EEG microstates is low with respect to typical fMRI-RSNs and the activity of single EEG microstates seems to correlate with activity of multiple networks^15^. Moreover, because of the low-spatial resolution of the EEG, volume conduction and source mixing could affect the topographical patterns of the microstates making this correspondence more complicated. Therefore, other approaches, such as decomposition of EEG time-frequency spectrum using NNMF^60^, could be used to reveal similar property of the data shading new light on the “idling-priors” theory of the simultaneous brain activity. Finally, here we preferred consistency of extraction and labeling methodology across conditions to subject-customized procedures, potentially affecting states variability. In the future, more robust methods of microstates extraction should be envisioned to reduce this variability.

In summary, identification of brain plasticity markers can aid in the prediction of functional outcome and the development of therapeutic interventions to support and promote recovery in motor disorders^9^. Spontaneous brain activity features could provide rich and sensitive markers^61,62^ for neuro-motor diseases in which motor abilities and dexterous hand movements are often lost or significantly reduced^8^. However, the definition of sensible and personalized biomarkers entails the identification of subject-specific features involved in the planning and execution of motor behaviors.

Previous studies showed that differences in microstates occurrence and duration correlate with altered brain functions in psychiatric conditions^30,41–45^. Here we demonstrated that differences in the dynamics of intrinsic coherent brain patterns revealed by subject-specific EEG microstate analysis are linked with motor control strategies, which are a known biomarker of motor deficits^63–65^. Therefore, subject-specific EEG microstates analysis could be particularly useful to describe and monitor the effect of motor training on cortical plasticity and brain functional re-organization when cortical networks are damaged, such as in stroke^66–68^. Indeed, EEG-based measures are safe, inexpensive, and accessible in complex medical settings and the high temporal resolution of EEG may be particular salient in studies of the motor system^9^.

Finally, the high decoding accuracies that we observed, especially when compared to EEG decoding performances (see ref.^53^ for a detailed summary of grasp decoding studies), are significant not only as evidence of a role of spontaneous brain activity in the generation and organization of motor behaviors, but also for brain-machine interfaces and neuro-feedback applications^69–71^. Indeed, we here showed (Supplementary Figure S5) that high decoding accuracy was achieved also when using only part of the data to extract the EEG microstates, envisaging the possibility to use microstate decoding in real-time.

Despite conceptual and technological challenges, we believe that multi-modal integration of non-invasive cortico-motor signals will foster the understanding of the impact of sensorimotor disorders on brain dynamics and boost the development of personalized neurorehabilitation protocols.

## Material and Methods

### Participants and experimental protocol

Eight right-handed healthy young subjects (6 males and 2 females, age range [23 29]) were enrolled in the study. The Brain & Mind Institute’s Ethics Committee for Human Behavioral Research of the École Polytechnique Fédérale de Lausanne approved the experiment, and the recordings were carried out in agreement with the Declaration of Helsinki. Written informed consent, for both study participation, and publication of identifying information and images, was obtained from all subjects.

#### Resting State

At the beginning and at the end of the experiment, the brain activity during resting state with eyes closed was recorded for 5 minutes.

#### Reaching/Grasping Tasks

Subjects were either asked to execute pure planar reaching movements or to reach, grasp, and hold 16 different objects with four different grasp types: ulnar, pulp, and five-finger pinch, and cylindrical grasp (see Table 1 and Supplementary Material). We choose reaching-and-grasping movements because they are one of the pivotal ways human interact with the world and they are often disrupted in neurological disorders.

**Table 1:**
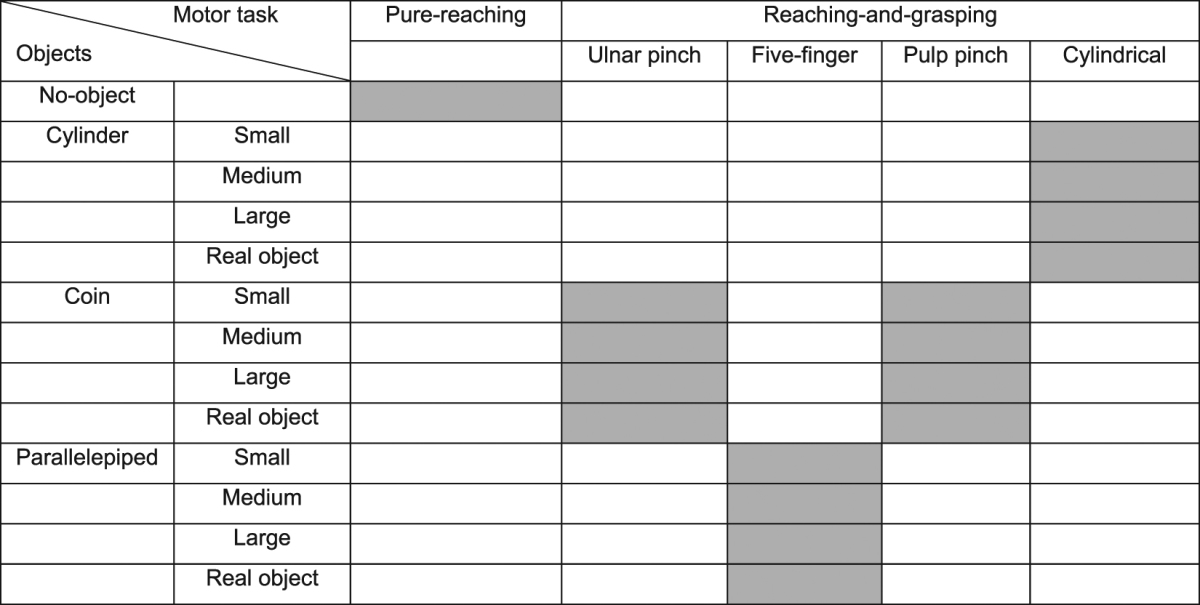
Combinations of motor tasks and objects. Grey squares indicate that an object was grasped with the corresponding grasp type.

The subjects seated on a chair with their right arm relaxed on the table. In the starting position, the elbow was flexed of about 90°, the upper arm was parallel to the trunk, and the right hand was placed on a starting button with the palm downward and the fist open. The object was placed on the table, in front of the subject, 30 cm far from the border of the table closest to the subject. The object’s position was fixed across objects, repetitions, and grasp types. During task execution, the subjects were instructed to keep their eyes fixed on the object’s position.

A monitor was used to indicate the experimental timeline (Fig. 1a): task presentation (0.5 seconds); 2 seconds pause; movement execution phase (about 1 second); 2 seconds holding phase (the appearance of a red dot on the monitor instructed the subject to release the object); return to the starting position (i.e., return phase, about 1 second); and 5 seconds pause before the beginning of a new task. At the end of the return phase, the experimenter manually changed the object. Videos recorded during the experiment were analyzed offline to check the positioning of the correct object by the experimenter and the performance of the correct grasp by the subject. We used contact sensors to record task events, such as the initiation of the movement and the beginning of the holding phase, and we recorded these signals simultaneously in EEG and EMG data for signal synchronization. The experimental timeline was controlled by an application implemented in LabVIEW (National Instrument) according to the information provided by the contact sensors. Task times were estimated from the sensors data as the time elapsed between task presentation and initiation of the movement (i.e., movement preparation time), and between movement onset and beginning of the holding phase (i.e., movement execution time).

Each task (pure-reaching or reaching-and-grasping for each object) was repeated 15 times. All movements were executed at a comfortable speed, and the object’s order and the grasp types were randomized.

Before starting the experiment, a test for the maximum voluntary contraction (MVC) of each muscle was performed.

Movement preparation and execution had a similar duration across participants and tasks (on average 4.10 ± 0.12 sec and 1.14 ± 0.13 sec for movement preparation and execution, respectively, see Table 2). Therefore, interactions between movement duration and microstates dynamics were not explored.

**Table 2:** Reaction time.

	Task presentation - movement onset	Movement onset - holding onset
Reaching	4.05 (0.13) sec	0.93 (0.13) sec
Ulnar	4.18 (0.16) sec	1.28 (0.14) sec
Five-finger	4.06 (0.11) sec	1.20 (0.12) sec
Pulp	4.11 (0.10) sec	1.20 (0.13) sec
Cylindrical	4.09 (0.11) sec	1.08 (0.11) sec

Time between task presentation and movement onset (left column) and between movement and holding onset (right column) for pure-reaching movements (top row) and for the four grasp types (ulnar pinch, five-finger pinch, pulp pinch, and cylindrical grasp). The average and the standard error refer to eight subjects.

The datasets generated and analyzed during the current study are available from the corresponding author on reasonable request.

### Data acquisition and pre-processing

EEG data were continuously acquired using a 64 channels Active-Two system with standard 10–20 configuration (Biosemi, Amsterdam, Netherlands) at a sampling rate of 2048 Hz.

EMG signals of 15 muscles from the upper and forearm and the hand (Table 3) were recorded by using superficial Ag-AgCl electrodes (Kendall H124SG, ECG electrodes 30 × 24 mm) after appropriate skin preparation with a Noraxon Desktop DTS wireless system at a sampling rate of 3 kHz.

**Table 3:** Recorded EMG.

	Muscle
1	trapezius superior (TRAP)
2	anterior deltoid (DANT)
3	biceps brachii long head (BICL)
4	triceps brachii long head (TRIC)
5	pronator teres (PRON)
6	flexor digitorum superficialis (FLDS)
7	extensor digitorum communis (EXDC)
8	flexor carpi ulnaris (FLCU)
9	extensor carpi ulnaris (EXCU)
10	flexor carpi radialis (FLCR)
11	extensor carpi radialis longus (EXCL)
12	flexor pollicis brevis (FLPB)
13	extensor pollicis brevis (EXPB)
14	adductor pollicis transversus (ADPT)
15	abductor digiti minimi (ABDM)

EEG and EMG data were preprocessed offline using MATLAB (MathWorks, Natick MA) and EEGLAB toolbox^72^.

The raw EEG data were filtered (1 Hz to 40 Hz, zero-phase IIR filters^11,12,73^), down-sampled at 128 Hz, and re-referenced to a common average. ‘Eye blink’ and ‘eye movements’ artifacts were removed by Independent Component Analysis (ICA)^72^.

Epochs were extracted for each object from 2 seconds before the beginning of the movement until the grasping of the object^37,74^, in case of reaching-and-grasping tasks, or until the reaching of the target position, in case of pure-reaching tasks (movement phase). Only 2 seconds before the beginning of the movement (i.e., 2 seconds of the entire movement preparation) were considered to avoid including the inter-trial interval in the epochs. Epochs for the holding phase were extracted from the grasping of the object or the reaching of the target position to 2 seconds after it. All epochs were visually inspected to discard those containing artifacts.

Epochs related to each grasp type and pure-reaching movements were averaged separately for the movement execution and the holding phase (i.e., in total 10 dataset for each subject)^75^. The selectivity of pre-motor neurons is determined not by the object shape but by the grip posture used to grasp the object^76^. Thus, objects grasped with the same grasps were averaged together. Before averaging, epochs were time interpolated at the same length using cubic splines over a number of points determined as the mean number of samples across epochs for pure-reaching and for each grasp type separately. The whole movement phase, i.e., 2 seconds of the entire movement preparation plus the time of the movement execution (about 1 second), and the whole holding phase (i.e., 2 seconds after object’s grasp) were used for microstates extraction.

For the resting state condition, we concatenated both dataset recorded before and after the experiment. Periods contaminated by artifacts were removed^10^. One minute of data, selected around the center of the 10 minutes segment, was used for microstates extraction.

The raw EMG signals were detrended, high-pass filtered at 50 Hz (Butterworth filter, 7th order), rectified, low-pass filtered with a cut-off frequency of 10 Hz (Butterworth filter, 7th order)^63^, and normalized for the MVC. Time epochs for the movement phase and the holding phase (the same used in the EEG analysis) were pooled together separately for each tasks (each grasp type and pure-reaching movements, i.e., in total 10 dataset for each subject).

### Extraction of subject-specific EEG microstates

Microstates extraction results in the decomposition of the brain activity in stable topographies allowing comparison across conditions.

For each subject and dataset (i.e., resting state, pure-reaching for movement and holding phase, and each grasp type for movement and holding phase), subject-specific EEG microstates were extracted independently, using a modified spatial cluster k-means algorithm implemented in CARTOOL^77^, with rejection of segments shorter than 23 ms and polarity of the topographical maps ignored. The optimal number of clusters (i.e., microstates) was set by seeking for the absolute minimum of the cross-validation function for each subject and dataset^77^. The same number of microstates was retained for all subjects in the same condition (i.e., mean number of microstates across subjects) to allow an easy intra-task comparison.

For the purpose of summarizing the results across all subjects, subject-specific microstates were matched across conditions. Specifically, for each condition, the subject-specific microstates were submitted to a second k-means cluster analysis, with the restriction that the microstates of each subject had to be classified into different clusters and with the number of clusters fixed to the one obtained in the first k-means cluster analysis. Subject-specific microstates within the same cluster were labeled with the same label. While this procedure ensured that similar microstates were labeled with a single convention, all the individual microstates were then used for subject-specific motor task analysis, independently of their ordering (see Section *Correlation* *between* *subject*-*specific* *microstates* *and* *motor* *control* *strategies*).

To identify the presence of spontaneous brain activity during motor behavior, microstates of motor tasks conditions (i.e., pure-reaching and each grasp type), both for movement and holding phases, were matched to the resting-state microstates correlating the most and the non-matching microstates (i.e., Pearson correlation below 0.65) were then compared across motor tasks.

### Extraction of subject-specific muscle synergies

For each subject and dataset independently (i.e., pure-reaching for movement and holding phase, and each grasp type for movement and holding phase), subject-specific muscle synergies were extracted by utilizing the L2-norm non-negative matrix factorization algorithm^36^ (NNMF). Results were consistent when using the KL divergence NNMF algorithm (see Supplementary Figure S2c,d). Due to non-convexity, each extraction was repeated 50 times, and the repetition with the solution explaining the highest overall amount of EMG variance was selected. The number of retained synergies was the number at which the variance accounted for (VAF) was higher than 98%^78,79^. The same number of muscle synergies was retained for all subjects to allow an easy intra-task comparison (i.e., mean number of muscle synergies across subjects).

Muscle synergies were matched across subjects and conditions according to their similarity (determined by using normalized scalar products, DOT, across muscle weights vectors) with a set of reference synergies. Muscle synergies were considered dissimilar if DOT < 0.65. The set of reference synergies was obtained for each condition by grouping the muscle synergies of all subjects with a hierarchical clustering procedure based on minimization of the Minkowski distance between muscle weights vectors^63^.

We evaluated muscle synergies dynamics by calculating muscle synergies temporal occurrence. The latter was estimated by computing the correlation between a putative EMG dataset, which was obtained by combining the synergy’s timing activation vector with the correspondent muscle weights vector, and the original EMG data. For each subject, temporal occurrence was then obtained averaging the correlation over epochs, considering windows of 100ms for movement planning (i.e., from minus 1 second to movement onset) and holding phase, and windows of 10% of the total movement duration for the movement phase (about 100ms).

### Time-frequency analysis

To verify that the EEG signals used to extract the microstates during movements contained features commonly identified as characteristic of motor-related brain activity (e.g., beta modulation before movement initiation and after object grasp^37,38^), time frequency spectra were calculated for each motor task from 1.5 second before movement onset to 1.5 second after object grasp using sliding Hamming windows of 200 ms with time steps of 32ms. For each subject, epoch, and frequency bin, power spectra were normalized for the average spectra in the corresponding frequency bin before averaging across epochs.

Comparison with EEG microstates was based on the averaged time frequency spectra over 7 electrodes of interest (Cz, CPz, Pz, C1, C3, C2, C4) covering the centro-parietal zone where the modulation of the beta power is maximal^37^. Specifically, we computed the most frequent microstate across epochs and subjects evaluated at the specific time points corresponding to desynchronization and synchronization of the beta-band identified from the averaged time frequency spectra over 7 electrodes of interest.

### Subject-specific EEG microstate dynamics

We evaluated EEG microstate dynamics by computing EEG microstate occurrences. The latter was obtained by computing the histogram of the most prevalent microstate within each temporal window for each epoch and subject independently. The temporal windows of the histogram were set to 100ms for movement planning (i.e., from minus 2 seconds to movement onset) and holding phase, and to windows of 10% of the total movement duration for the movement execution phase (about 100ms). For the time windows in which two or more microstates had the same highest prevalence, we assigned as prevalent microstate the one present in the previous time window. This approach replicates the frequently used temporal smoothing^77^. In the case in which the microstate assigned in the previous time window or in the following window was not one of the two (or more) with the highest prevalence in the examined window, the microstate assigned was chosen randomly between the two (or more) with equivalent prevalence.

We calculated significant differences across motion phases (i.e., between 2 seconds of the entire movement preparation and movement execution, and between movement execution and holding phase), and across motor tasks for movement preparation, movement execution, and holding phase separately. For each comparison, the significance threshold was obtained from a null-distribution constructed randomly permuting the microstates occurrence values of the two conditions compared. The number of permutations was determined to have α = 0.05.

### Correlation between subject-specific microstates and motor control strategies

In order to quantify whether microstate occurrences might reflect differences in motor control strategies and, thus, a functional role of spontaneous brain activity in the generation of volitional motor behavior in humans, we *i)* calculated a multivariate analysis of correlation, i.e., canonical correlation^46^, between all elements of the set of microstates and those of the muscle synergies set, and *ii)* employed a Bayesian classifier, specifically a Linear Discriminant Analysis (LDA)^80^ to reveal the unique correspondence between microstates occurrence and motor task performed.

For reaching-and-grasping movements, canonical correlation was computed for each subject independently combining occurrences of microstates and muscle synergies for the four grasp types and the three motion phases (i.e., 2 seconds of the entire movement preparation, movement execution, and holding phase) together. For pure-reaching movements, instead, canonical correlation was computed for each subject independently using microstates and muscle synergies occurrence for the three motion phases. The significance of the canonical components was obtained with a one-sample non-parametrical t-test over subjects’ correlation values.

For each subject and grasp type independently, a four-class LDA classifier was built using half of the epochs of that grasp and the same amount of epochs for the other three grasps as training set (84.38 +/− 7.87 epochs). The remaining epochs of that grasp type were used as test data (21 +/− 2 epochs). The feature vectors used for the LDA classifier consisted of the microstate occurrences over movement preparation. This corresponded to a feature vector of dimension 100, i.e., 20 time windows for 5 microstates (i.e., B, C, D, E, and F). For each subject independently, the classification was repeated 1000 times shuffling training and testing sets (cross-validation). Decoding accuracy values were averaged over repetitions. We calculated the mean-standard deviation ratio of the LDA coefficients over the 1000 repetitions (maximum value 1.85) and we assessed statistical significance of these ratios versus the 95^th^ percentile of a null-distribution (48% of coefficients were statistically significant). The null distribution was estimated repeating the LDA 10000 times for each subject with labels of the training set randomly shuffled at each repetition. Significance decoding accuracy level corresponded to the average accuracy across repetitions and subjects. LDA was performed also using only the resting-state microstates’ occurrence or only the task-related microstates’ occurrence over movement preparation as feature vectors. In both cases, before performing LDA, the influence of the occurrence of a microstates set (i.e., resting-state or task-related microstates set) was removed from that of the other set using linear regression. Then, the four-class LDA classifier was built following the same procedure aforementioned.

### Statistical procedures

Subject-specific microstates and muscle synergies were used in the analysis. In order to summarize the information, the results reported were averaged across subjects. All data are reported as mean values +/− standard error of the mean. Significance was analyzed using non-parametric Wilcoxon rank sum test (α = 0.05, Bonferroni corrected) for the temporal characterization of the microstates (see Supplementary Materials), and permutation approach for the microstates occurrence and to determine chance level of the LDA.

## Electronic supplementary material

Supplementary materials

## References

[1] Fox MD, Raichle ME (2007). Spontaneous fluctuations in brain activity observed with functional magnetic resonance imaging. Nature Reviews Neuroscience.

[2] Fox MD (2005). The human brain is intrinsically organized into dynamic, anticorrelated functional networks. Proceedings of the National Academy of Sciences of the United States of America.

[3] Greicius MD, Supekar K, Menon V, Dougherty RF (2009). Resting-state functional connectivity reflects structural connectivity in the default mode network. Cerebral cortex.

[4] Ghosh A, Rho Y, McIntosh AR, Kötter R, Jirsa VK (2008). Noise during rest enables the exploration of the brain’s dynamic repertoire. PLoS Comput Biol.

[5] Deco G, Corbetta M (2011). The dynamical balance of the brain at rest. The Neuroscientist.

[6] Spadone S (2015). Dynamic reorganization of human resting-state networks during visuospatial attention. Proceedings of the National Academy of Sciences.

[7] Betti V (2013). Natural scenes viewing alters the dynamics of functional connectivity in the human brain. Neuron.

[8] Rossini PM, Calautti C, Pauri F, Baron J-C (2003). Post-stroke plastic reorganisation in the adult brain. The Lancet Neurology.

[9] Wu J (2015). Connectivity measures are robust biomarkers of cortical function and plasticity after stroke. Brain.

[10] Koenig T (2002). Millisecond by millisecond, year by year: normative EEG microstates and developmental stages. Neuroimage.

[11] Britz J, Van De Ville D, Michel CM (2010). BOLD correlates of EEG topography reveal rapid resting-state network dynamics. Neuroimage.

[12] Van de Ville D, Britz J, Michel CM (2010). EEG microstate sequences in healthy humans at rest reveal scale-free dynamics. Proceedings of the National Academy of Sciences.

[13] Britz, J., Hernàndez, L. D., Ro, T. & Michel, C. M. EEG-microstate dependent emergence of perceptual awareness. *Frontiers in behavioral neuroscience* **8** (2014). 10.3389/fnbeh.2014.00163 PMC4030136 24860450

[14] Musso F, Brinkmeyer J, Mobascher A, Warbrick T, Winterer G (2010). Spontaneous brain activity and EEG microstates. A novel EEG/fMRI analysis approach to explore resting-state networks. Neuroimage.

[15] Yuan H, Zotev V, Phillips R, Drevets WC, Bodurka J (2012). Spatiotemporal dynamics of the brain at rest—exploring EEG microstates as electrophysiological signatures of BOLD resting state networks. Neuroimage.

[16] Bizzi E, Tresch MC, Saltiel P, d’Avella A (2000). New perspectives on spinal motor systems. Nature reviews. Neuroscience.

[17] Bizzi E, Cheung V, d’Avella A, Saltiel P, Tresch M (2008). Combining modules for movement. Brain research reviews.

[18] Bernstein, N. *The coordination and regulation of movement*. (Pergamon, 1967).

[19] Ivanenko YP, Poppele RE, Lacquaniti F (2004). Five basic muscle activation patterns account for muscle activity during human locomotion. The Journal of physiology.

[20] Pirondini E (2016). Evaluation of the effects of the Arm Light Exoskeleton on movement execution and muscle activities: a pilot study on healthy subjects. Journal of NeuroEngineering and Rehabilitation.

[21] Dominici N (2011). Locomotor primitives in newborn babies and their development. Science.

[22] Ivanenko YP, Cappellini G, Dominici N, Poppele RE, Lacquaniti F (2007). Modular control of limb movements during human locomotion. The Journal of neuroscience: the official journal of the Society for Neuroscience.

[23] Tresch MC, Saltiel P, Bizzi E (1999). The construction of movement by the spinal cord. Nature neuroscience.

[24] Bizzi E, Cheung VC (2013). The neural origin of muscle synergies. Frontiers in computational neuroscience.

[25] Mussa–Ivaldi FA, Bizzi E (2000). Motor learning through the combination of primitives. Philosophical Transactions of the Royal Society of London B: Biological Sciences.

[26] Overduin SA, d’Avella A, Roh J, Carmena JM, Bizzi E (2015). Representation of Muscle Synergies in the Primate Brain. The Journal of Neuroscience.

[27] Leo A (2016). A synergy-based hand control is encoded in human motor cortical areas. eLife.

[28] Rana M, Yani MS, Asavasopon S, Fisher BE, Kutch JJ (2015). Brain Connectivity Associated with Muscle Synergies in Humans. The Journal of Neuroscience.

[29] Minguillon, J. *et al*. In *Engineering in Medicine and Biology Society (EMBC), 2014 36th Annual International Conference of the IEEE*. 2093–2096 (IEEE). 10.1109/EMBC.2014.6944029 25570397

[30] Lehmann D (2005). EEG microstate duration and syntax in acute, medication-naive, first-episode schizophrenia: a multi-center study. Psychiatry Research: Neuroimaging.

[31] Mantini D, Perrucci MG, Del Gratta C, Romani GL, Corbetta M (2007). Electrophysiological signatures of resting state networks in the human brain. Proceedings of the National Academy of Sciences.

[32] Smith SM (2009). Correspondence of the brain’s functional architecture during activation and rest. Proceedings of the National Academy of Sciences.

[33] Cole MW (2013). Multi-task connectivity reveals flexible hubs for adaptive task control. Nature neuroscience.

[34] Dominici N (2009). Changes in the limb kinematics and walking-distance estimation after shank elongation: evidence for a locomotor body schema?. Journal of neurophysiology.

[35] d’Avella A, Portone A, Fernandez L, Lacquaniti F (2006). Control of fast-reaching movements by muscle synergy combinations. The Journal of neuroscience: the official journal of the Society for Neuroscience.

[36] Lee DD, Seung HS (2001). Algorithms for non-negative matrix factorization. Adv Neur In.

[37] Zaepffel M, Trachel R, Kilavik BE, Brochier T (2013). Modulations of EEG beta power during planning and execution of grasping movements. PloS one.

[38] Jurkiewicz MT, Gaetz WC, Bostan AC, Cheyne D (2006). Post-movement beta rebound is generated in motor cortex: evidence from neuromagnetic recordings. Neuroimage.

[39] Spinks RL, Kraskov A, Brochier T, Umilta MA, Lemon RN (2008). Selectivity for grasp in local field potential and single neuron activity recorded simultaneously from M1 and F5 in the awake macaque monkey. The Journal of Neuroscience.

[40] Pistohl T, Schulze-Bonhage A, Aertsen A, Mehring C, Ball T (2012). Decoding natural grasp types from human ECoG. Neuroimage.

[41] Koenig T (1999). A deviant EEG brain microstate in acute, neuroleptic-naive schizophrenics at rest. European archives of psychiatry and clinical neuroscience.

[42] Kikuchi M (2011). EEG microstate analysis in drug-naive patients with panic disorder. PLoS One.

[43] Kindler J, Hubl D, Strik W, Dierks T, Koenig T (2011). Resting-state EEG in schizophrenia: auditory verbal hallucinations are related to shortening of specific microstates. Clinical Neurophysiology.

[44] Nishida K (2013). EEG microstates associated with salience and frontoparietal networks in frontotemporal dementia, schizophrenia and Alzheimer’s disease. Clinical Neurophysiology.

[45] Andreou C (2014). Resting-state connectivity in the prodromal phase of schizophrenia: insights from EEG microstates. Schizophrenia research.

[46] Hardoon DR, Szedmak S, Shawe-Taylor J (2004). Canonical correlation analysis: An overview with application to learning methods. Neural computation.

[47] Milz P (2016). The functional significance of EEG microstates—Associations with modalities of thinking. NeuroImage.

[48] Weiss EJ, Flanders M (2004). Muscular and postural synergies of the human hand. Journal of neurophysiology.

[49] Breteler MDK, Simura KJ, Flanders M (2007). Timing of muscle activation in a hand movement sequence. Cerebral Cortex.

[50] Winges SA, Santello M (2004). Common input to motor units of digit flexors during multi-digit grasping. Journal of neurophysiology.

[51] Miller, K. J. *et al*. Human motor cortical activity is selectively phase-entrained on underlying rhythms. (2012). 10.1371/journal.pcbi.1002655 PMC3435268 22969416

[52] Schwab S (2015). Discovering frequency sensitive thalamic nuclei from EEG microstate informed resting state fMRI. NeuroImage.

[53] Agashe, H. A., Paek, A. Y., Zhang, Y. & Contreras-Vidal, J. L. Global cortical activity predicts shape of hand during grasping. *Frontiers in neuroscience* **9** (2015). 10.3389/fnins.2015.00121 PMC4391035 25914616

[54] Nir Y (2008). Interhemispheric correlations of slow spontaneous neuronal fluctuations revealed in human sensory cortex. Nature neuroscience.

[55] Biswal B, Zerrin Yetkin F, Haughton VM, Hyde JS (1995). Functional connectivity in the motor cortex of resting human brain using echo‐planar mri. Magnetic resonance in medicine.

[56] Greicius MD, Krasnow B, Reiss AL, Menon V (2003). Functional connectivity in the resting brain: a network analysis of the default mode hypothesis. Proceedings of the National Academy of Sciences.

[57] Hipp JF, Hawellek DJ, Corbetta M, Siegel M, Engel AK (2012). Large-scale cortical correlation structure of spontaneous oscillatory activity. Nature neuroscience.

[58] Brookes MJ (2011). Measuring functional connectivity using MEG: methodology and comparison with fcMRI. Neuroimage.

[59] Baker AP (2014). Fast transient networks in spontaneous human brain activity. Elife.

[60] Lee H, Cichocki A, Choi S (2009). Kernel nonnegative matrix factorization for spectral EEG feature extraction. Neurocomputing.

[61] Greicius MD, Srivastava G, Reiss AL, Menon V (2004). Default-mode network activity distinguishes Alzheimer’s disease from healthy aging: evidence from functional MRI. Proceedings of the National Academy of Sciences of the United States of America.

[62] Filippini N (2009). Distinct patterns of brain activity in young carriers of the APOE e4 allele. Neuroimage.

[63] Cheung VC (2009). Stability of muscle synergies for voluntary actions after cortical stroke in humans. Proc Natl Acad Sci USA.

[64] Cheung VC (2012). Muscle synergy patterns as physiological markers of motor cortical damage. Proc Natl Acad Sci USA.

[65] Tropea P, Monaco V, Coscia M, Posteraro F, Micera S (2013). Effects of early and intensive neuro-rehabilitative treatment on muscle synergies in acute post-stroke patients: a pilot study. Journal of neuroengineering and rehabilitation.

[66] Honey CJ, Sporns O (2008). Dynamical consequences of lesions in cortical networks. Hum. Brain Mapp..

[67] Alstott J, Breakspear M, Hagmann P, Cammoun L, Sporns O (2009). Modeling the impact of lesions in the human brain. PLoS Comput Biol.

[68] Khanna A, Pascual-Leone A, Michel CM, Farzan F (2015). Microstates in resting-state EEG: current status and future directions. Neuroscience & Biobehavioral Reviews.

[69] Varkuti B (2013). Resting state changes in functional connectivity correlate with movement recovery for BCI and robot-assisted upper-extremity training after stroke. Neurorehabilitation and Neural Repair.

[70] Buch E (2008). Think to move: a neuromagnetic brain-computer interface (BCI) system for chronic stroke. Stroke.

[71] Hernandez LD, Rieger K, Baenninger A, Brandeis D, Koenig T (2016). Towards using microstate-neurofeedback for the treatment of psychotic symptoms in schizophrenia. A feasibility study in healthy participants. Brain Topography.

[72] Delorme A, Makeig S (2004). EEGLAB: an open source toolbox for analysis of single-trial EEG dynamics including independent component analysis. Journal of neuroscience methods.

[73] Dipietro, L., Poizner, H. & Krebs, H. I. Spatiotemoral Dynamics of Online Motor Correction Processing Revealed by High-density Electroencephalography. (2014). 10.1162/jocn_a_00593 PMC4692805 24564462

[74] Van Wijk B, Daffertshofer A, Roach N, Praamstra P (2009). A role of beta oscillatory synchrony in biasing response competition?. Cerebral Cortex.

[75] Gindrat A-D (2015). Whole-scalp EEG mapping of somatosensory evoked potentials in macaque monkeys. Brain Structure and Function.

[76] Umilta MA, Brochier T, Spinks RL, Lemon RN (2007). Simultaneous recording of macaque premotor and primary motor cortex neuronal populations reveals different functional contributions to visuomotor grasp. Journal of neurophysiology.

[77] Brunet D, Murray MM, Michel CM (2011). Spatiotemporal analysis of multichannel EEG: CARTOOL. Computational intelligence and neuroscience.

[78] Clark DJ, Ting LH, Zajac FE, Neptune RR, Kautz SA (2010). Merging of healthy motor modules predicts reduced locomotor performance and muscle coordination complexity post-stroke. Journal of neurophysiology.

[79] Ting LH, McKay JL (2007). Neuromechanics of muscle synergies for posture and movement. Current opinion in neurobiology.

[80] Fisher RA (1936). The use of multiple measurements in taxonomic problems. Annals of eugenics.

