# Supplementary material for: EEG topographies provide subject-specific correlates of motor control
Source: Sci Rep. 2017 Oct 16;7:13229. doi: 10.1038/s41598-017-13482-1 (PMC5643537; doi:10.1038/s41598-017-13482-1)
Supplement: Supplementary file 1 — Supplementary materials [file 41598_2017_13482_MOESM1_ESM.pdf]

Supplementary Information

EEG topographies provide subject-specific correlates of motor control

Elvira Pirondini, Martina Coscia, Jesus Minguillon, José del R. Millán, Dimitri Van De Ville[+], Silvestro Micera[+]

Supplementary material and methods

Motor tasks details

Subjects were asked to execute pure planar reaching movements or to reach, grasp, and hold 16 different objects with four different grasp types: ulnar and pulp pinch (precision bidigital grasps), and five-finger pinch and cylindrical grasp (power multifinger grasps). Grasps and objects (a key, a coin, a small box, and a bottle) were chosen to mimic everyday life movements[1]. Additional nine objects were used to account for small, medium, and large grips: three coins (diameter: 0.02 m, thickness: 0.005 m, 0.01 m, and 0.015 m, respectively), three parallelepipeds (base: 0.1x0.02 m, height: 0.02 m, 0.04 m, and 0.06 m, respectively), and three cylinders (height: 0.1 m, diameter: 0.02 m, 0.04 m, and 0.06 m, respectively). The different sizes were introduced to avoid habituation of the participants to the task.

Temporal characteristics of EEG microstates

To characterize and compare EEG microstates across different conditions, mean microstate duration, mean number of microstates per second, and percentage of the total analysis time covered by each microstate[2] were computed. Values were calculated for each subject and epoch. Subject-specific values were obtained averaging across epochs and were compared between conditions using Wilcoxon rank sum test ($\alpha=0.05$) Bonferroni corrected for the number of comparisons (i.e., 15 for microstates A, B, C, and D, 10 for microstate E, and 6 for microstate F)[3].

Statistical quantification of EEG microstate dynamics during resting state

We evaluated EEG microstate dynamics by computing EEG microstate occurrences.

The latter was obtained by calculating the histogram of the most prevalent microstate within each temporal window (100ms) for each subject independently. In order to statistically quantify whether there was a modulation of EEG microstate dynamics over time, we divided the resting state period (i.e., one minute) in sliding windows of 2 seconds. We calculated significant differences between consecutive windows. For each comparison (i.e., comparison between consecutive windows), the significance threshold was obtained from a null-distribution constructed randomly permuting the microstates occurrence values of the two temporal windows compared. The number of permutations was determined to have $\alpha=0.05$.

Additional LDA analysis

In the manuscript, we employed a Bayesian classifier, specifically a Linear Discriminant Analysis (LDA)[4], to reveal the unique correspondence between microstates occurrences and motor task performed. Here we extended this analysis. Specifically, we tested decoding accuracy *i)* when extracting microstates only in the training epochs, and *ii)* when using only movement execution phase as feature (in this case the dimension of the feature vector was 45, i.e., 9 time windows per 5 microstates). For the second test, we used the EEG microstates extracted averaging the signal over all epochs (i.e., microstates reported in the main manuscript).

For the first test, we selected a single repetition of the cross-validation procedure. We extracted microstates for each grasp and subject independently for the averaged signals over the training epochs (i.e., half of the epochs of each grasp randomly chosen). The extracted microstates were then used to evaluate EEG microstate occurrences for both dataset (i.e., testing and training). The testing dataset corresponded to the remaining epochs not used to extract the microstates. For each subject and grasp type independently, a four-class LDA classifier was built using the microstates occurrences of the training epochs, and it was tested using the microstates occurrences of the testing epochs. As previously, the feature vectors

used for the LDA classifier consisted of the microstate occurrences over movement preparation.

Supplementary results

Temporal characteristics of the resting-state microstates are preserved

We assessed differences across conditions by evaluating the microstates temporal characteristics (i.e., mean duration, mean number of microstates, and total time covered, Figure S1).

The four resting-state EEG microstates showed a slightly lower mean duration than the ones of age-matched (25-30 years old) healthy subjects reported in literature[2]. However, the mean number of microstates and the total time covered were comparable with previous results for all microstates[2].

In general, during movement execution and holding phase, all the rest-specific microstates (i.e., A, B, C, and D) showed similar temporal characteristic compared to resting state. Only, microstate C, for pure-reaching movements and for power grasp (i.e., five-finger pinch and cylindrical grasp), and microstate D, for reaching-and-grasping movements, showed a reduced frequency of occurrence during the movement phase (Wilcoxon test, $p<0.003$). In addition, microstates A and C had a significant shorter total time covered for pure-reaching during the movement phase (Wilcoxon test, $p<0.003$).

No significant differences were found across motor tasks, grasp types, and between movement and holding phases, except for microstate C.

Figure S1 around here

Muscle synergies are consistent between L2-norm and KL divergence algorithm

In the manuscript, for each subject and dataset independently, subject-specific muscle synergies were extracted by utilizing the L2-norm NNMF algorithm[5]. Here, we

checked consistency of results when using the KL divergence NNMF algorithm. Also for KL divergence we found that five (4.88±0.29 across subjects and motor tasks) and four (4.03± 0.42 across subjects and motor tasks) muscle synergies were sufficient to reconstruct more than 98% of the variance in the original signals respectively for movement execution and holding phase (see Figure S2**c**). The muscle weights of the synergies were highly similar between the two algorithms (mean DOT=0.98 and DOT=0.94 for movement execution and holding phase, respectively). Also the matching across motor tasks was preserved when using the KL algorithm. Indeed, synergies Syn 1, 2, and 3 were common across motor tasks (mean DOT=0.89). The fourth synergy (Syn 4), instead, was grasping-specific (mean DOT=0.97 across grasp types and mean DOT=0.44 between pure-reaching and reaching-and-grasping). Surprisingly the fifth synergy (Syn 5), which represented the contribution of the finger flexors and was not present in the holding phase except in pure-reaching, was substituted in five-finger pinch by an additional synergy (Syn 6) for the control of the thumb (mean DOT=0.65).

Figure S2 around here

Microstates prediction of motor task is preserved when using only part of the data to extract them

In both tests (i.e., microstate extractions for training epochs and movement execution phase prediction), the decoding accuracy obtained (70% and 62% for the first and second test, respectively) was comparable to the one attained when extracting microstates in averaged signals containing both training and testing epochs during movement preparation.

Figure S5 around here

Figures

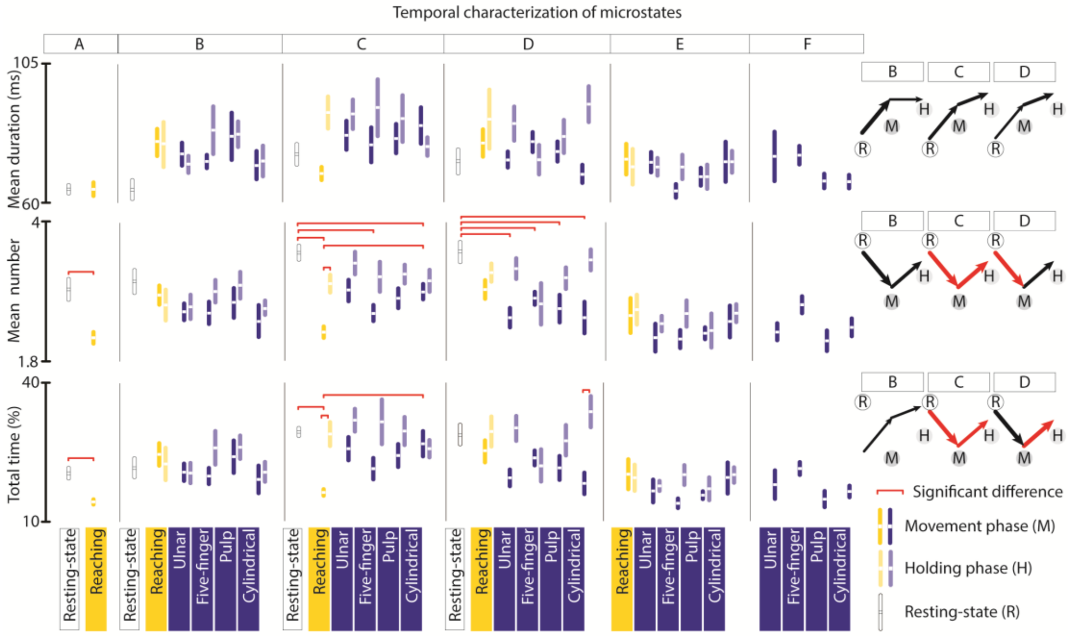

Figure S1. Temporal characterization of EEG microstates. To characterize and compare EEG microstates across different conditions, mean microstate duration (left, top row), mean number of microstates per second (left, middle row), and percentage of the total analysis time covered (left, bottom row) were computed for each microstate, subject, and epoch. Subject-specific values were obtained averaging across epochs. For each condition and microstate, the rectangles are proportional to average value ± standard error across subjects, and they are centered on the average values. Subject-specific values were compared across conditions with a Wilcoxon rank sum test (α=0.05) Bonferroni corrected for the number of comparisons. Red lines indicate significant differences across conditions. Right: Schematic summary of the results. Line thickness codes the number of motor tasks presenting that specific behavior. Arrows pointing down represent a decrease in value between two conditions (i.e., resting state (R), movement phase (M), and holding phase (H)), while arrows pointing up represent an increase in value between two conditions. Black and red lines code non-significant and significant differences (Wilcoxon rank sum test, significant level α=0.05, Bonferroni corrected) between two conditions, respectively.

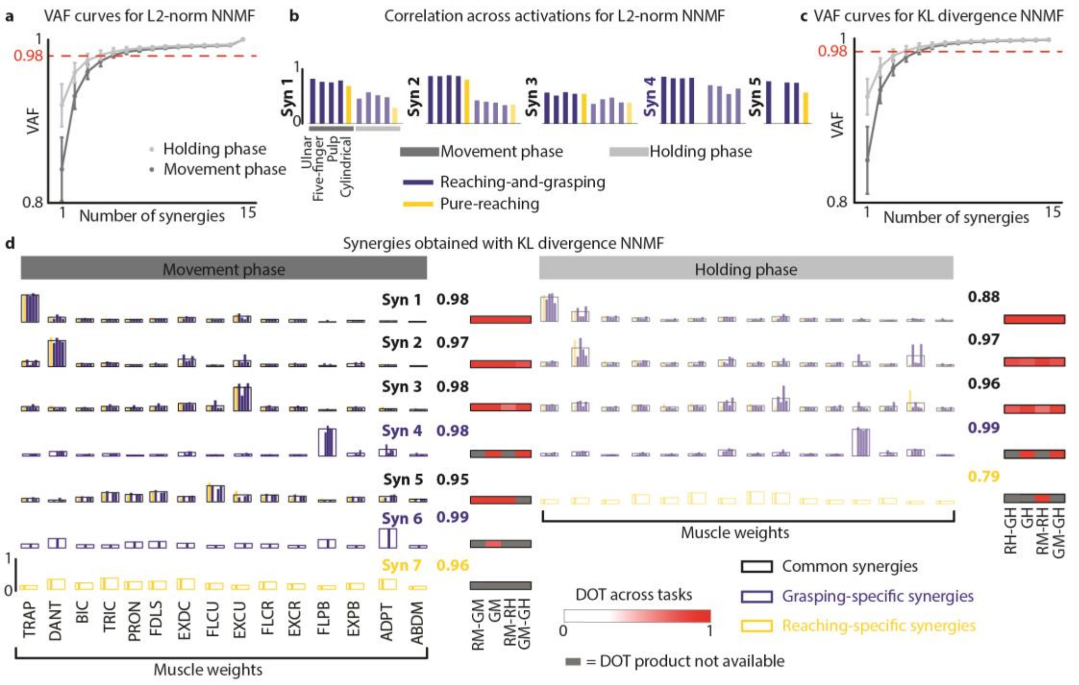

Figure S2. Muscle synergies. (a) VAF curves averaged over subjects and motor tasks for L2-norm NNMF algorithm. (b) For each synergy, bar plots indicate average (over subjects and conditions) Pearson correlation of the activation coefficients of each motor task with the other motor tasks. (c) VAF curves averaged over subjects and motor tasks for KL divergence NNMF algorithm. (d) Subject-specific muscle synergies were extracted using the KL divergence NNMF algorithm for each subject and motor task independently. Muscle synergies were matched among subjects and conditions according to their similarity with a set of reference synergies. Left panels: muscle weights vectors for each reference synergy during movement phase. Right panels: muscle weights vectors for each reference synergy during holding phase. For synergies common across motor tasks (i.e., Syn 1, 2, 3, and 5), blue and yellow bars show the weight coefficients for each grasp type (blue bars) and for pure-reaching (yellow bar). Black bar profiles indicate means across motor tasks. For grasping-specific synergies (i.e., Syn 4 and 6), blue bars show the weight coefficients for each grasp type and blue bar profiles indicate means across grasp types. The numbers in

bold are the mean DOT values between muscle synergies found with the L2-norm NNMF algorithm and those obtained with the KL divergence algorithm. DOT values across motor tasks are reported for each synergy using red levels: RM-GM and RH-GH are the DOT products between pure-reaching and reaching-and-grasping during movement and holding phase, respectively. GM and GH are the average DOT products across grasp types during movement and holding phase, respectively; RM-RH and GM-GH are the DOT products between movement and holding phase for pure-reaching and reaching-and-grasping, respectively. RM indicates pure-reaching during movement phase; GM indicates reaching-and-grasping during movement phase; RH indicates pure-reaching during holding phase; GH indicates reaching-and-grasping during holding phase. Grey squares code “DOT product not available" (e.g., for Syn 4 comparisons between reaching-and-grasping and pure-reaching is not possible because Syn 4 was not present during pure-reaching).

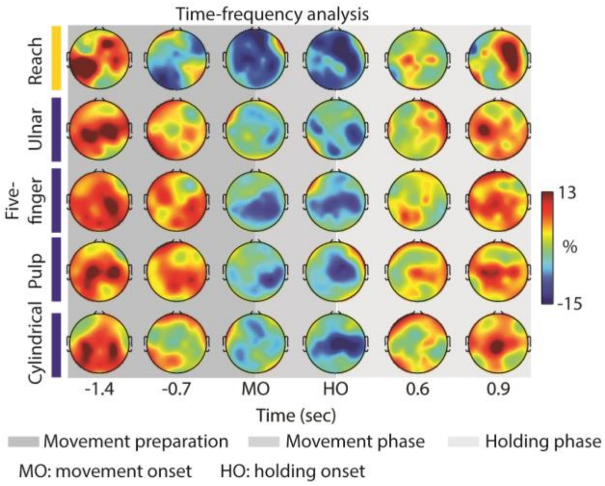

Figure S3. Time-frequency analysis. Time frequency spectra were calculated for each subject, epoch, and motor task from 1.5 second before movement onset (MO) to 1.5 second after object grasp (i.e., holding onset, HO). The plots display topographical maps (grand average across subjects and epochs) of the beta power spectra (20Hz) expressed in percentages compared to the average spectrum (0%) as a function of time for each motor task.

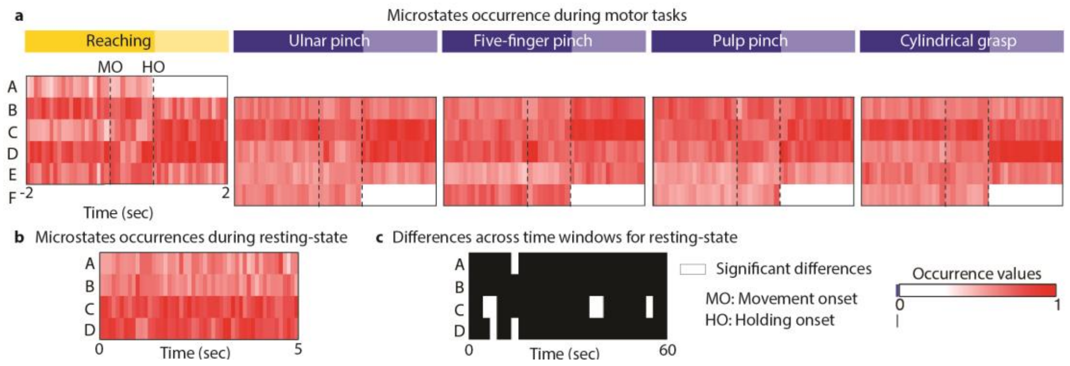

Figure S4. EEG microstates dynamics. We evaluated EEG microstate dynamics by computing EEG microstates occurrences for each subject independently. (a) Average (across subjects) EEG microstates occurrences for pure-reaching and each grasp type separately are coded in red (range [0 1]). The values of the histogram are normalized over time and microstates. Black dashed lines code movement (MO) and holding onset (HO). (b) The EEG microstates occurrence for 5 seconds resting state averaged over subjects is coded in red (range [0 1]). The values of the histogram are normalized over time and microstates. (c) Statistical quantification of microstates occurrences over time windows of 2 seconds for one-minute of resting state. White rectangles code time windows in which the microstate occurrences were significantly different from the previous time window. Microstates occurrences were significantly different between consecutive time windows only in few cases (7% of the cases). Therefore, we can conclude that the presence of microstates was not modulated over time during resting state.

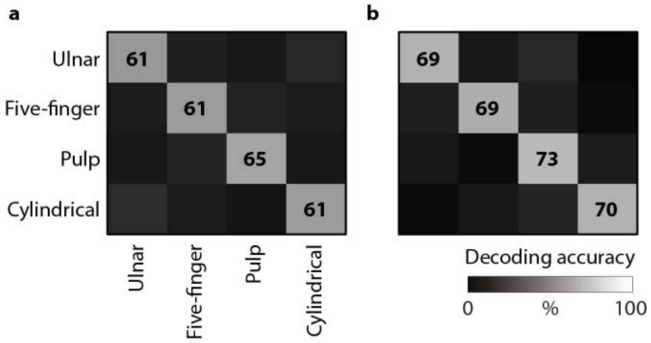

Figure S5. Additional LDA results. We tested the decoding accuracy when extracting the EEG microstates using only the training epochs **(a).** Confusion matrices for the four grasp types were averaged over subjects. Grey levels ([0 100%]) code the decoding accuracy values. We then tested the decoding accuracy when using only movement execution phase as feature **(b)**. Confusion matrices for the four grasp types were averaged over subjects and cross-validation repetitions.

References

1 Sollerman, C. & Ejeskär, A. Sollerman hand function test: a standardised method and its use in tetraplegic patients. *Scandinavian Journal of Plastic and Reconstructive Surgery and Hand Surgery* **29**, 167-176 (1995).

2 Koenig, T. *et al.* Millisecond by millisecond, year by year: normative EEG microstates and developmental stages. *Neuroimage* **16**, 41-48 (2002).

3 Brodbeck, V. *et al.* EEG microstates of wakefulness and NREM sleep. *Neuroimage* **62**, 2129-2139 (2012).

4 Fisher, R. A. The use of multiple measurements in taxonomic problems. *Annals of eugenics* **7**, 179-188 (1936).

5 Lee, D. D. & Seung, H. S. Algorithms for non-negative matrix factorization. *Adv Neur In* **13**, 556-562 (2001).
